# Interplay between RNA viruses and cGAS/STING axis in innate immunity

**DOI:** 10.3389/fcimb.2023.1172739

**Published:** 2023-04-03

**Authors:** Lucia Amurri, Branka Horvat, Mathieu Iampietro

**Affiliations:** Centre International de Recherche en Infectiologie (CIRI), Centre International de Recherche en Infectiologie, Team Immunobiology of Viral infections, Univ Lyon, Inserm, CNRS, Université Claude Bernard Lyon 1, Ecole Normale Supérieure de Lyon, Lyon, France

**Keywords:** cGAS/STING, RNA virus, innate immunity, viral evasion, host-pathogen interactions, antiviral strategies

## Abstract

While the function of cGAS/STING signalling axis in the innate immune response to DNA viruses is well deciphered, increasing evidence demonstrates its significant contribution in the control of RNA virus infections. After the first evidence of cGAS/STING antagonism by flaviviruses, STING activation has been detected following infection by various enveloped RNA viruses. It has been discovered that numerous viral families have implemented advanced strategies to antagonize STING pathway through their evolutionary path. This review summarizes the characterized cGAS/STING escape strategies to date, together with the proposed mechanisms of STING signalling activation perpetrated by RNA viruses and discusses possible therapeutic approaches. Further studies regarding the interaction between RNA viruses and cGAS/STING-mediated immunity could lead to major discoveries important for the understanding of immunopathogenesis and for the treatment of RNA viral infections.

## 1 Introduction

### 1.1 cGAS/STING signalling pathways and its activators

**Figure 1:**
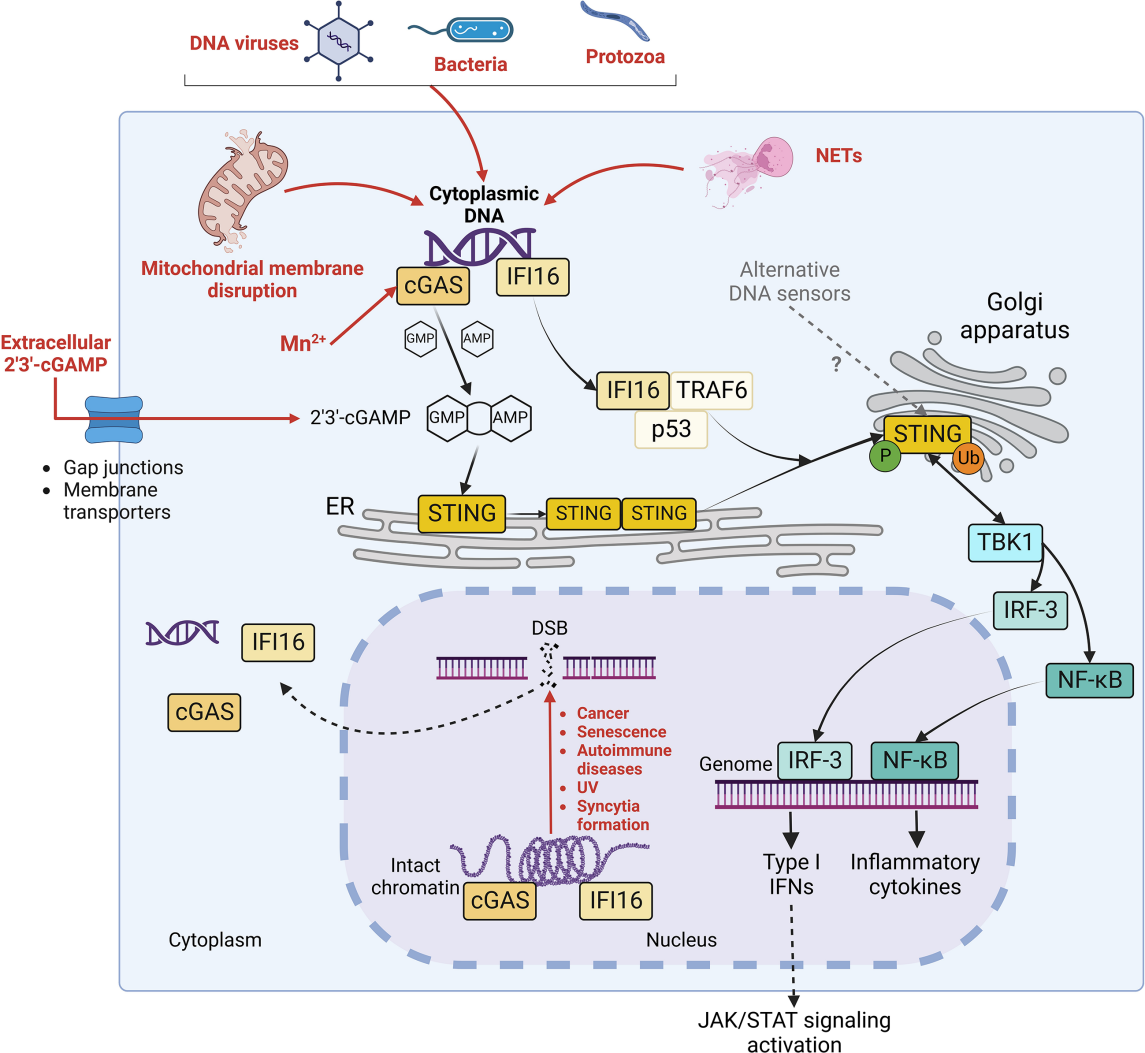
STING signalling pathways. Diverse stimuli (in red), including infections by DNA virus, bacteria or protozoa, mitochondrial membrane disruption, possible Mn^2+^ release, extracellular cGAMP import and neutrophil extracellular traps (NETs) generation trigger the release of intracellular DNA in the cytoplasm. Moreover, double strand breaks (DSB) in genomic DNA, occurring during cancer, senescence, autoimmune disorders, UV exposure and membrane fusion, contribute to the liberation of self-DNA in cytosol and to the dissociation of cGAS and IFI16 (in yellow) from intact chromatin. Both cGAS and IFI16 detect cytoplasmic DNA (in purple). However, while cGAS activates STING by synthesizing its agonist 2’3’-cGAMP, IFI16 elicits STING activation through a non-canonical mechanism in complex with p53 and TRAF6. Once activated, STING oligomerizes and translocates to Golgi apparatus, where it undergoes phosphorylation and ubiquitination. Consequently, it stimulates the nuclear translocation of IRF-3 and NF-kB transcription factors and further expression of IFN I and inflammatory cytokines, leading to the activation of JAK/STAT signaling pathway in autocrine and paracrine manner.

Cyclic guanosine monophosphate-adenosine monophosphate (cyclic GMP-AMP, cGAMP) synthase (cGAS)/stimulator of interferon (IFN) genes (STING) pathway was identified in 2008 as the major signalling axis of innate immune response responsible for the sensing of cytosolic double-strand DNA (dsDNA) (Ishikawa and Barber, 2008). At basal state, dsDNA is confined in enclosed cellular compartments but gains access to cytoplasm during certain perturbations of the cellular homeostasis. This could occur during infection by DNA viruses, bacteria and protozoa, where microbial DNA directly serves as pathogen-associated molecular pattern (PAMP) to trigger cGAS/STING activation. In addition, host DNA can represent a damage-associated molecular pattern (DAMP) responsible for STING activation after the disruption of nuclear and/or mitochondrial membrane integrity taking place under stress conditions and in cancerous, senescent or infected cells (**Figure 1**) (Hopfner and Hornung, 2020).

The monomeric form of the sensor cGAS mediates cytoplasmic DNA sequence-independent recognition by binding B-form dsDNA minor groove through a conserved zinc-ion-binding domain (Civril et al., 2013). Then, cGAS dimerizes and oligomerizes, catalysing the synthesis of the second messenger cGAMP through the cyclization of guanosine triphosphate (GTP) and adenosine triphosphate (ATP) (Xia et al., 2016) (**Figure 1**). cGAMP activates the endoplasmic reticulum (ER)-resident STING by inducing a conformational change which triggers its dimerization and consequent oligomerization (Ergun et al., 2019). The assembly of oligomers requires the palmitoylation of STING at C88 and C91 by DHHC3, 7 and 15 palmitoyltransferases, which is crucial to allow STING translocation from ER to Golgi network and perinuclear puncta (Mukai et al., 2016). In the Golgi apparatus, STING undergoes S366 phosphorylation by TANK-binding kinase 1 (TBK1) (Dobbs et al., 2015; Tsuchiya et al., 2016, 1) and non-degradative K63-linked ubiquitination by tripartite motif containing protein 32 (TRIM32) at K224, by TRIM56 at K150 and by tumor necrosis factor receptor-associated factor 6 (TRAF6) E3 ubiquitin ligases (Tsuchida et al., 2010, 56; Zhang et al., 2012, 32; Balka et al., 2020). Phosphorylated STING (p-STING) dimers serve as a docking site for IFN regulatory factor 3 (IRF-3), which is therefore phosphorylated at two sites (S386/385 and S396-S405) by TBK1 (Panne et al., 2007; Tanaka and Chen, 2012; Dobbs et al., 2015; Liu et al., 2015; Yum et al., 2021). Active IRF-3 transcription factor then translocates to the nucleus, where it interacts with IFN-stimulated response element (ISRE), triggering type I IFN (IFN-I) expression and further IFN-stimulated genes (ISG) transcription through Janus kinase-signal transducer and activator of transcription (JAK-STAT) signalling (Schindler et al., 2007; Dobbs et al., 2015). In parallel, K63-linked ubiquitinated STING preferentially stimulates pro-inflammatory cytokines expression through a TBK1/IKKϵ-NF-κB phosphorylation cascade (Balka et al., 2020).

Interestingly, even though cGAS was thought to reside exclusively in cytoplasm, recent studies reported a more complex situation, with cGAS shuttling between cytoplasm, plasma membrane and nucleus (Volkman et al., 2019; Michalski et al., 2020; Herzner et al., 2021) (**Figure 1**). In the nucleus, cGAS is either sequestered in an inactive state through its interaction with intact chromatin or in an active state in presence of DNA structure perturbations (Michalski et al., 2020; Herzner et al., 2021; MacDonald et al., 2022). This suggests that the recognition of nuclear DNA by cGAS could be regulated by additional mechanisms, involving modifications in chromatin availability and/or unknown nuclear factors modulating the catalytic activity of cGAS (Michalski et al., 2020; MacDonald et al., 2022). Moreover, it has been reported that manganese (Mn^2+^), which is is required as an enzymatic cofactor in many physiologic processes and released in the cytosol from mitochondria and Golgi network upon stress conditions, is a direct agonist of cGAS, as it triggers conformational change and the synthesis of cGAMP even in absence of cytosolic DNA (Wang et al., 2018; Zhao et al., 2020). Interestingly, Mn^2+^ has been recently shown to inhibit both RNA and DNA virus infections in a cGAS/STING axis-independent manner as well, suggesting a redundancy in the possible antiviral mechanisms stimulated by Mn^2+^ (Sun et al., 2023). Finally, cGAS detects neutrophil extracellular traps (NETs), chromatin-based structures released by neutrophils during regulated neutrophil death (NETosis) when combatting microbes (Apel et al., 2021). When neutrophils are engulfed by macrophages as a mechanism of resolution of an infection, NETs translocate to the cytoplasm of macrophages, where they trigger cGAS/STING signalling activation (Apel et al., 2021).

In addition to cGAS, other cytoplasmic and/or nuclear DNA sensors contribute to the activation of STING signalling, with possible redundancy and cell specificity (Ma and Damania, 2016) (**Figure 1**). Like cGAS, IFNγ inducible protein 16 (IFI16) traffics between nucleus and cytoplasm (Unterholzner et al., 2010; Dunphy et al., 2018). It binds nuclear damaged DNA independently of its sequence through its DNA-binding hematopoietic expression-IFNγ inducible-nuclear localization (HIN) domain, together with ataxia-telangiectasia mutated (ATM) and poly(ADP-ribose) polymerase 1 (PARP-1) DNA repair proteins (Aguilar-Quesada et al., 2007; Goubau et al., 2010; Goubau et al., 2007). This interaction triggers the translocation of IFI16 to the cytosol, where it activates STING through a cGAMP-independent mechanism in complex with p53 tumor suppressor and TRAF6 ubiquitin ligase (Dunphy et al., 2018). Moreover, while more DNA sensors have been identified to date, such as DEAD-box helicase-41 (DDX41) (Zhang et al., 2011, 4), DNA-dependent activator of IFN-regulatory factors (DAI) (Takaoka et al., 2007), IFIX (Diner et al., 2015), DNA-protein kinase (DNA-PK) (Ferguson et al., 2012), DExH-box helicase 9 (DHX9) (Kim et al., 2010), DHX36 (Kim et al., 2010), DDX60 (Miyashita et al., 2011, 60) and MRE11 (Kondo et al., 2013, 11), their role in STING signalling regulatory network requires further investigations to be completely elucidated. Finally, in addition to the direct synthesis of cGAMP by cGAS, STING signalling can be activated by cGAMP which is packaged into viral particles and exchanged between bystander cells in order to induce an antiviral environment and prevent infections (Bridgeman et al., 2015; Gentili et al., 2015). Furthermore, the transfer of cGAMP into the cell occurs through either gap junctions or membrane transporters, such as leucine-rich repeat-containing 8/volume regulated anion channel (LRRC8/VRAC), folate transporter solute carrier family 19 member 1 (SLC19A1) or ATP-gated channel P2X 7 purinergic Receptor (P2X7R) (Ablasser et al., 2013; Luteijn et al., 2019; Ritchie et al., 2019; Zhou et al., 2020a ; Zhou et al., 2020b).

### 1.2 Innate immune response to RNA virus infections by canonical RNA-sensing pathways

**Figure 2:**
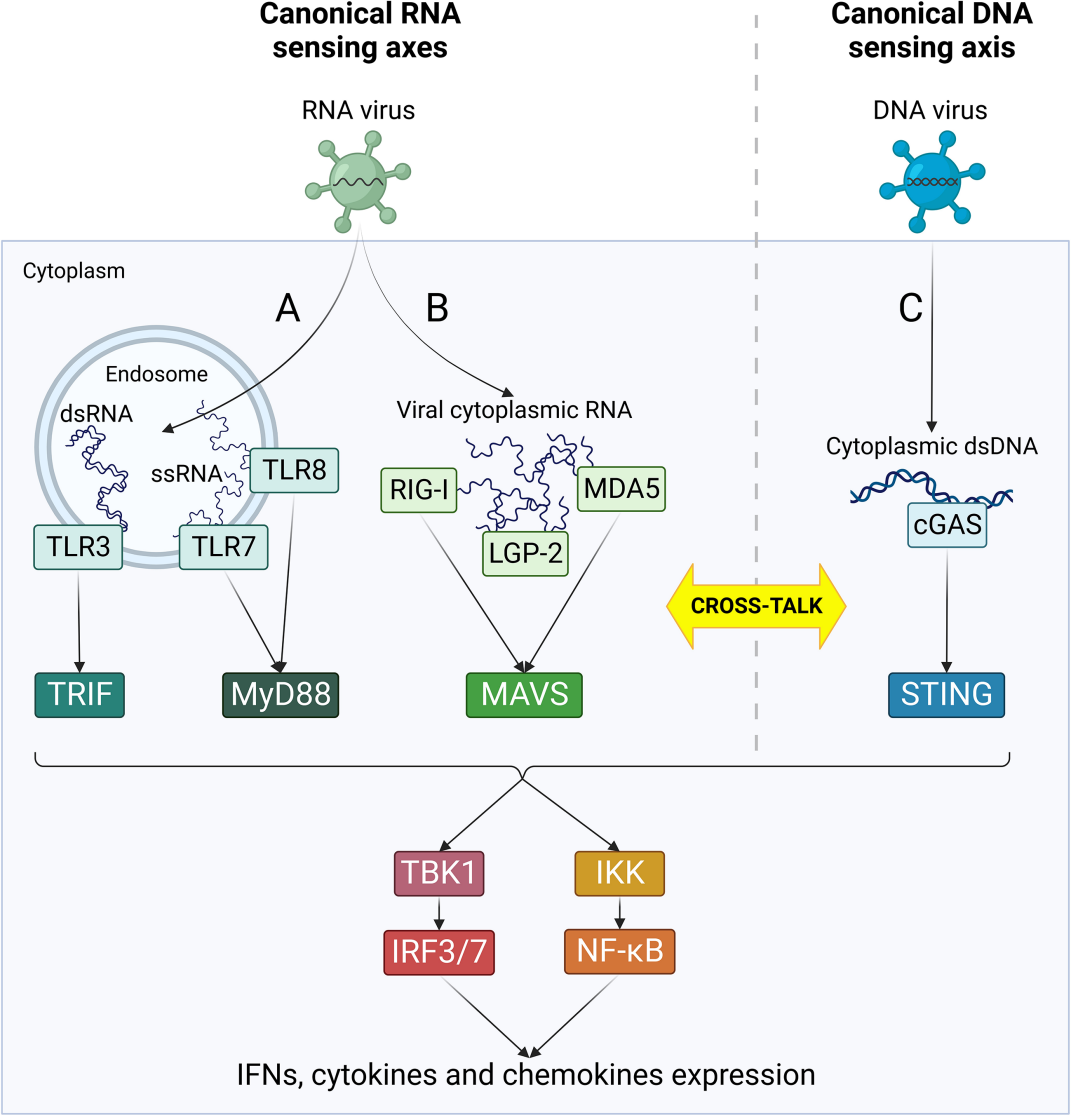
Canonical RNA- and DNA-sensing pathways. Following viral penetration into target cells, viral nucleic acids are recognized by cellular PRRs specialized in the detection of specific RNA and DNA species. RNA-sensing pathways (in green) are mediated by TLRs **(A)** and RLRs **(B)** and can sense different types of virus-derived RNA in endosomes or cytoplasm, respectively. **(C)** cGAS/STING is the major cytoplasmic DNA-sensing axis (in blue) and it plays an essential role as primary player in the canonical response to DNA virus infections. According to each receptor activation, different adaptor molecules are recruited, leading to the formation of four separate innate immune axes: TRL3/TRIF, TLR7/8/MyD88, RLRs/MAVS and cGAS/STING. Nonetheless, all these pathways converge in an unique cascade with the activation of TBK1/IRF3/7 and IKK/NF-kB, ultimately resulting in the activation of IFN response and cytokines production. Moreover, the four axes can cross-talk at different levels both directly and indirectly, leading to a complex network of interactions between the effectors of innate immune response.

Following RNA virus infections, viral RNA is rapidly detected by cellular pattern recognition receptors (PRRs), including Toll-like receptors (TLRs) and retinoic acid inducible gene-I (RIG)-like receptors (RLRs) (Xu et al., 2021a) (**Figure 2**). TLR3 and 7/8 are localized in endosomal compartments and are responsible for the recognition of endosomal double strand and single strand RNA, respectively (Kawasaki and Kawai, 2014). TLR3 signals through TIR domain-containing adaptor inducing IFN-β (TRIF) adaptor protein, while TLR7 and 8 recruit Myeloid differentiation primary response 88 (Myd88) (Kawasaki and Kawai, 2014). In parallel, cytoplasmic viral RNA is detected by RLRs, namely RIG-I, melanoma differentiation-associated gene 5 (MDA5) and laboratory of genetics and physiology 2 (LGP-2) (Rehwinkel and Gack, 2020). All these three receptors contain a central helicase domain responsible for RNA binding. However, just RIG-I and MDA5 are capable to initiate a downstream signaling through their caspase activation and recruitment domains (CARD), while LGP-2 acts as immune regulator by inhibiting RIG-I and enhancing MDA5 function (Rehwinkel and Gack, 2020). Following interaction with immunostimulatory RNA, RIG-I and MDA5 engage mitochondrial antiviral signaling protein (MAVS) localized on the outer mitochondrial membrane. TRIF-, MyD88-, MAVS- and STING-dependent signaling cascades all converge in the activation of IKKα/β-NF-κB and TBK1-IRF3/7 axes, leading to the rapid expression of IFN type I and III, cytokines and chemokines and the induction of ISG through JAK/STAT signaling (Xu et al., 2021a). The superimposition of downstream immune effectors allows a quick amplification of signals and witnesses a deep interconnection between these four signaling axes.

### 1.3 Cross-talk between RNA- and DNA-sensing innate immune signaling pathways

Numerous cross-talk mechanisms between RNA and DNA sensing pathways exist and play an important role in the response to viral infections (Cai et al., 2021) (**Figure 2**). First, RLRs participate in the response to DNA virus infections, such as Epstein Barr virus (EBV) and Herpes simplex virus-1 (HSV-1), through the recognition of dsRNA transcribed from the viral genome (Chiu et al., 2009; Rasmussen et al., 2009). In addition, host-derived RNAs can contribute to RLR-dependent IFN induction, as observed during KSHV infection (Zhao et al., 2018). Mutual activation between RNA and DNA sensing axes can also occur. TRIF binds STING and favors its activation during HSV-1 infection, and STING expression is induced by RIG-I (Liu et al., 2016; Latif et al., 2020). In turn, STING can bind to both RIG-I and MAVS and amplify their downstream signals thanks to its localization in mitochondria-associated ER membrane (MAM) (Zhong et al., 2008; Nazmi et al., 2012). Finally, IFI16 can directly bind Influenza A virus (IAV) and Chikungunya virus (CHIKV) RNA, thus emerging as a novel RNA binding protein (RBP) in addition to its canonical role as DNA sensor (Kim et al., 2020; Jiang et al., 2021). Moreover, IFI16 promotes RIG-I activation by two different mechanisms: it triggers K63-linked ubiquitination of RIG-I and recruits RNA Polymerase II on RIG-I promoter to induce it transcription (Jiang et al., 2021).

### 1.4 Non-canonical response to RNA virus infection by cGAS/STING axis

Thanks to its ability to sense both non-self and endogenous cytosolic and/or nuclear dsDNA, STING pathway plays a central role not only in immune response against microbial infections, but also in autoimmunity, inflammation, senescence and cancer, acting as a regulator of IFN-I and NF-κB expression (Motwani et al., 2019). While its involvement in the sensing of DNA viruses is well deciphered (Ishikawa et al., 2009; Lau et al., 2015), a contribution of STING signalling in the protection against RNA viruses has progressively emerged. The first evidence of its participation in the control of RNA virus infections came from the discovery of an antagonizing activity of Dengue virus (DENV) non-structural proteins over cGAS/STING (Aguirre et al., 2012; Aguirre et al., 2017). This observation was later extended to other flaviviruses, like Zika (ZIKV) (Kumar et al., 2016), West Nile (WNV) (McGuckin Wuertz et al., 2019), Hepatitis C (HCV) (Ding et al., 2013), Japanese encephalitis (JEV) (Nazmi et al., 2012) and Yellow Fever virus (YFV) (Ding et al., 2013), before being attributed to other viral families, such as *Coronaviridae* (Sun et al., 2012), *Orthomyxoviridae* (Holm et al., 2016), *Togaviridae* (Webb et al., 2020) and *Rhabdoviridae* (Rodríguez-García et al., 2018). Moreover, the activation of STING has been demonstrated after infection from various RNA viruses. Nonetheless, these viruses do not elicit any DNA intermediate step during their replication cycle, raising the question of how the DNA-related STING pathway can be activated during a RNA virus infection. While a direct activation of cGAS by viral RNA is not likely to occur, since RNA-cGAS interaction does not elicit cGAMP production (Yu and Liu, 2021), an indirect activation of cGAS/STING through uncharacteristic mechanisms has been demonstrated.

Among the putative mechanisms of indirect stimulation, the activation of STING by cross-talk with MAVS following RNA sensing by RLRs has been shown to occur upon some RNA virus infection, like Sendai virus (SeV), Vesicular stomatitis virus (VSV), Newcastle disease virus (NDV) and Japanese encephalitis virus (JEV) (Nazmi et al., 2012; Zevini et al., 2017). Moreover, it has been observed that STING signalling is triggered by endogenous DNA, namely nuclear or mitochondrial (mtDNA), leaking in cytoplasm following RNA virus infections (Hopfner and Hornung, 2020). DENV, Sars-CoV-2, Influenza A virus (IAV) and Measles virus (MeV) induce mitochondrial stress in infected cells *via* diverse mechanisms, causing mitochondrial membrane rupture and cytosolic release of mtDNA, further sensed by cGAS or alternative DNA sensors (Chatel-Chaix et al., 2016; Moriyama et al., 2019; Sato et al., 2021; Domizio et al., 2022). In parallel, the activation of cGAS/STING has been reported following chromatin damage, observed not only in cancerous and senescent cells, but also during infections by DNA, RNA viruses and bacteria (Pépin et al., 2017; Hopfner and Hornung, 2020) (**Figure 1**). DNA damage, like double-strand breaks (DSB), provokes the collapse of the nuclear envelope, leading to micronuclei formation, progressive increase of chromatin shattering and chromothripsis (Mackenzie et al., 2017; Kwon et al., 2020). Then, nuclear and/or cytoplasmic damaged chromatin binds and activates cGAS, as it has been observed in the context of *Coronaviridae* infections (Ren et al., 2021).

The leakage of both nuclear and mitochondrial DNA has been proposed to be subsequently elicited following virus-induced membrane fusion (Holm et al., 2012). Indeed, it was first reported that herpesvirus (HSV)-derived fusogenic virus-like particles (VLPs) induced a low grade IFN-I response and triggered a subset of IFN-stimulated genes (ISG), while fusion-deficient VLPs failed to trigger an innate immune response (Holm et al., 2012). In addition, it was reported that fusogenic liposomes, capable of triggering IFN-I expression, lost this ability only in mice deficient for STING and not for MyD88, TRIF and MAVS (Holm et al., 2012). These observations suggest a role of membrane fusion in the induction of a IFN-I response through a pathway independent of TLRs/RLRs and dependent on STING signalling (Maringer and Fernandez-Sesma, 2014). This hypothesis was then confirmed by the evidence that syncytia-inducing bacteria are able to trigger genomic instability, a danger signal which results in the formation of micronuclei, eventually activating cGAS/STING (Ku et al., 2020). The process of endogenous DNA release in the cytoplasm in response to membrane fusion has been hypothesized to be mediated by intracellular calcium influx subsequent to membrane structure perturbations (Maringer and Fernandez-Sesma, 2014). Indeed, it has been shown that VLPs-induced membrane fusion triggers phosphorylation and activation of protein kinase B (PKB/Akt) by the calcium-dependent phosphoinositide 3 kinase (PI3K) (Holm et al., 2012; Divolis et al., 2016). Thus, Ca^2+^, as a second messenger, may indirectly link cell membrane fusion and STING signalling activation through the induction of a further cellular damage, aimed at preventing viral spreading by triggering the activation of an anti-viral response and/or by inducing cell death (Holm et al., 2012).

## 2 Activation of STING by RNA viruses

**Figure 3:**
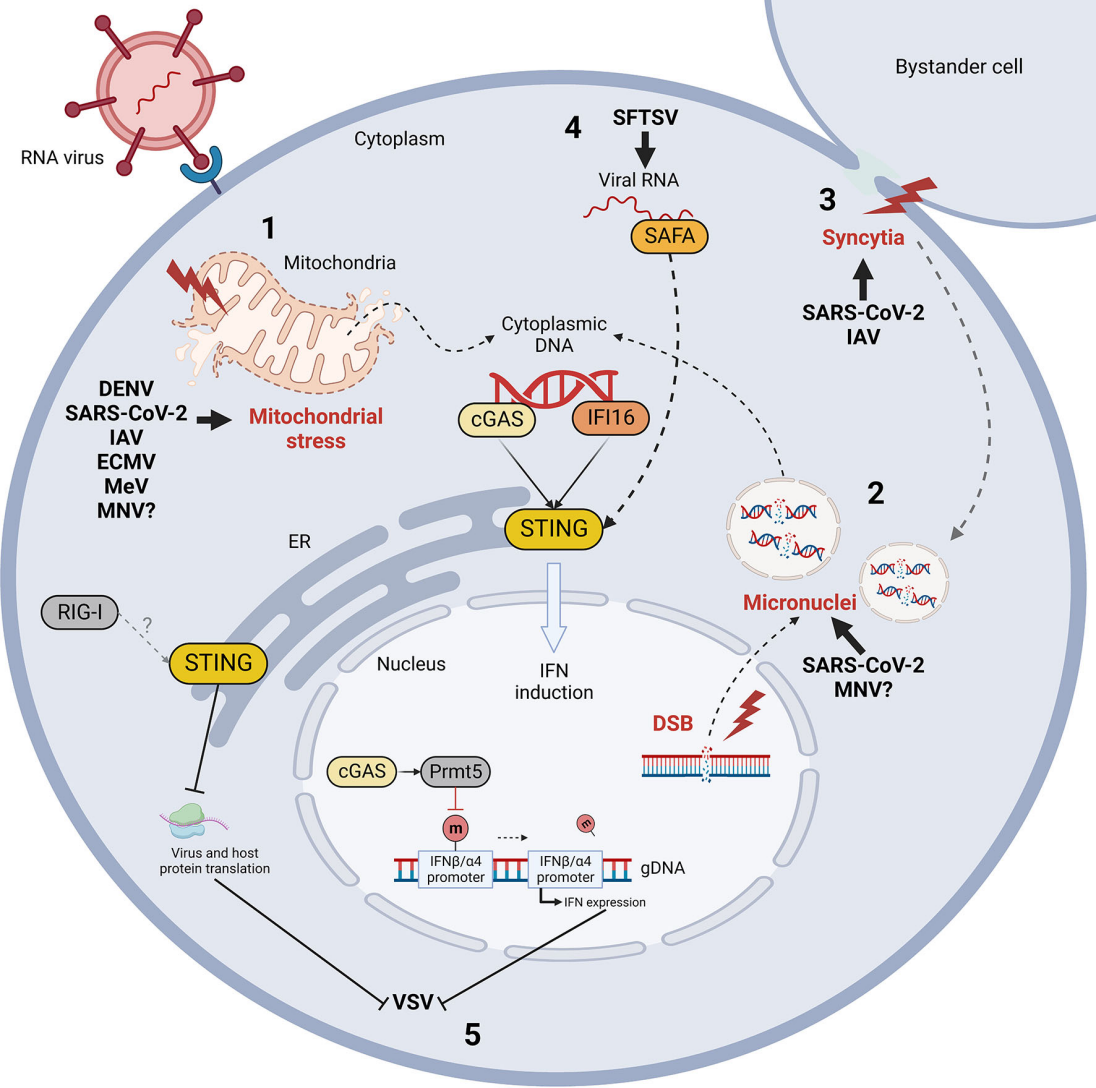
Mechanism of indirect cGAS/STING activation by RNA virus infection and STING-mediated control of RNA viral evasion. RNA virus infections trigger the release of self dsDNA in the cytoplasm due to mitochondrial stress (1) and/or genomic DNA damage and micronuclei formation (2) (red). Double strand breaks (DSB) and micronuclei can also be induced by virus-induced cell-to-cell fusion (syncytia formation, in red) (3). Cytoplasmic DNA is then detected by cGAS and IFI16 sensors leading to STING activation. Moreover, STING can be activated non-canonically by nuclear matrix protein scaffold attachment factor A (SAFA) through the mediation of viral RNA during *Bunyaviridae* infections (4). Finally, while IFN induction is considered the major STING-dependent anti-viral mechanism, STING and cGAS can also limit vesicular stomatitis virus (VSV) infection by inhibiting protein translation and by enhancing IFN expression through Prmt5 activation, respectively (5). DENV, Dengue virus; SARS-CoV-2, severe acute respiratory syndrome coronavirus 2; IAV, influenza A virus; ECMV, encephalomyocarditis virus; MeV, measles virus; MNV, murine norovirus; SFTSV, severe fever with thrombocytopenia syndrome virus; m, methyl group.

RNA virus infections elicit STING activation through diverse processes which share one common feature: the indirect and/or non-canonical activation of STING (**Figure 3**). This chapter describes the mechanisms of STING activation during RNA virus infections identified up to now, aiming at identifying similarities and distinctive features of different viral families.

### 2.1 Flaviviridae

While flaviviruses do not display any DNA-intermediate step during their replication cycle, they activate the DNA-sensing cGAS/STING pathway (Kao et al., 2018) along RLR RNA cellular sensors. This was originally suggested by the observation that STING silencing through siRNA induced a higher level of DENV viral RNA levels in primary human monocyte-derived dendritic cells (MDDCs), indicating an inhibitory role of STING pathway on DENV replication (Aguirre et al., 2012). A similar result was obtained for WNV, when the STING knock-out (KO) Goldenticket mice were proven to be more susceptible to WNV infection, resulting in increased morbidity and mortality compared to the WT animals (McGuckin Wuertz et al., 2019; Zhu and Fernandez-Sesma, 2020). Moreover, the fact that, among the euthanised mice, the STING KO ones displayed prevalently neurological symptoms, in opposition to the GI tract abnormalities exhibited by the WT, suggested the involvement of STING in the restriction of WNV from CNS (McGuckin Wuertz et al., 2019). Indeed, whereas intracranial WNV infection did not exert any significant difference in viral load between WT and Goldenticket mice, footpad infection induced a higher viral load in CNS in STING KO compared to WT mice, associated to increased lesions, mononuclear cells infiltration and neuronal death (McGuckin Wuertz et al., 2019). Furthermore, an aberrant T cell response, characterized by an altered CD4/CD8 T cell ratio (with decreased CD8^+^ and Treg cells and increased CD4^+^ cells) was observed in the spleen and the brain of STING KO mice (McGuckin Wuertz et al., 2019). These results suggest a role of the cGAS/STING signalling pathway in the balancing of cytotoxic and immunosuppressive adaptive response, fundamental for the control of WNV infection (Winkelmann et al., 2016).

The immunoprecipitation of cGAS and analysis of the cGAS-associated nucleic acid by qPCR and single-molecule real time sequencing (SMRT) revealed a significant enrichment of mtDNA bound to cGAS during DENV infection (Aguirre et al., 2017; Kao et al., 2018) (**Figure 3**). This, together with the morphological alteration of mitochondria, confirmed that an indirect activation of cGAS occurs upon DENV infection through the release of mtDNA in the cytoplasm due to mitochondrial stress (Chatel-Chaix et al., 2016). The proposed mechanism of mitochondria fragmentation involves DENV NS2B3, able to cleave mitofusins (MFN1 and 2), resulting in the disruption of the mitochondrial membrane potential (MMP) and consequent release of mtDNA in the cytosol, further aimed at impairing the mitochondria-associated RIG/MAVS signalling (Kao et al., 2018). Moreover, DENV structural M protein contributes to this process by binding mitochondrial membrane, causing permeabilization, matrix swelling and MMP loss (Catteau et al., 2003).

### 2.2 Coronaviridae

cGAS/STING activation has been characterized during SARS-CoV-2 infection and may play a central role in the pathogenesis of COVID-19 (Berthelot et al., 2020; Rui et al., 2021; Domizio et al., 2022). The involvement of STING signalling in *Coronaviridae* infections has been suggested initially by the fact that bats expressing a STING protein defective for anti-viral response induction do not display any disease manifestation, despite their capacity to harbour several coronaviruses (Xie et al., 2018; Berthelot et al., 2020; Mougari et al., 2022). One of the hallmarks of severe COVID-19 is an unbalanced immune response, with reduced IFN-I expression and overproduction of inflammatory cytokines causing systemic inflammatory symptoms (Cevik et al., 2020). Indeed, increased phosphorylation and nuclear accumulation of NF-κB p65, but not IRF3, is observed in SARS-CoV-2-infected cells (Neufeldt et al., 2020). This might be due to an anomalous activation of STING, that displays a defective translocation from ER to Golgi following SARS-CoV-2 infection, despite its re-localization to perinuclear regions (Neufeldt et al., 2020).

cGAS/STING activation following SARS-CoV-2 infection has been demonstrated to be triggered by syncytia formation through the interaction between the viral spike protein (S) and the cellular receptor angiotensin converting enzyme 2 (ACE2) (Ren et al., 2021) (**Figure 3**). Indeed, cell-to-cell fusion induces the production of micronuclei, where DNA damage and DNA damage response pathway (DDR) activation occur (Ren et al., 2021; Liu et al., 2022a). Micronuclear DNA colocalizes with cGAS in infected cells, triggering its activation, the subsequent phosphorylation of STING and IRF3 followed by the expression of IFNβ and ISGs (Ren et al., 2021; Liu et al., 2022a). Moreover cGAS is activated in endothelial cells by mtDNA released in the cytoplasm, as observed in lung-on-chip model and in skin biopsies from COVID-19 patients (Domizio et al., 2022).

### 2.3 Orthomyxoviridae

STING-dependent mediation of influenza virus appears to be essential for limiting virus replication *in vivo*, since STING KO mice display a significant increase in viral titers following IAV infection compared to WT mice (Moriyama et al., 2019). Contrarily, cGAS KO mice do not exhibit a higher viral titer in the lung compared to WT mice, suggesting a cGAS-independent STING activation during IAV infection (Moriyama et al., 2019). These results confirm previous observations that IFN-I production is significantly reduced in infected STING KO THP1 cells compared to WT cells, but not in cGAS KO THP1s (Holm et al., 2016). The cGAS-independent mechanism involved during IAV infection suggests a potential redundancy between different nucleic acid sensors capable of activating STING. Indeed, IFI16 has been shown to inhibit viral replication and sustain IFN-I production during IAV infection (Jiang et al., 2021). Both *in vitro* and *in vivo* models demonstrated that IFI16 is important in the protection against IAV in a non-canonical manner, by binding to both viral RNA and RIG-I. Moreover, IFI16 transcriptionally upregulates the expression of RIG-I and positively regulates its activation (Jiang et al., 2021). However, a possible IFI16-dependent STING activation during IAV infection should be investigated, as STING activation is observed also in absence of MAVS or cGAS, suggesting the presence of alternative activators of STING and its independency from RIG-I signalling (Holm et al., 2016).

Two distinct mechanisms seem to contribute to the activation of STING signalling pathway after IAV infection (Holm et al., 2016; Moriyama et al., 2019) (**Figure 3**). On one hand, cell-cell fusion observed during IAV infection may stimulate STING activation and IFN-I production through a cGAS-independent pathway (Holm et al., 2012; Holm et al., 2016; Zhang et al., 2022a). On the other hand, IAV M2 viroporin ion channel triggers the translocation of mtDNA into the cytosol in a MAVS-dependent manner, leading to the recognition of mtDNA by cGAS and subsequent activation of STING by cGAMP (Moriyama et al., 2019). The same phenomenon occurs for the 2B viroporin protein of encephalomyocarditis picornavirus (ECMV) (Moriyama et al., 2019). Furthermore, since viroporin-induced disturbance of intracellular ionic balance is responsible for Mn^2+^ efflux from cellular organelles, viral ion channels assembly may contribute to increase the sensitivity of cGAS to dsDNA or to activate cGAS in a DNA-independent manner (Wang et al., 2018; Moriyama et al., 2019; Zhao et al., 2020).

### 2.4 Paramyxoviridae

A contribution of the cGAS/STING axis in the protection from the negative-sense ssRNA Measles (MeV) and Nipah (NiV) paramyxoviruses has been recently pointed out (Iampietro et al., 2021). First, it was observed that mice KO for MyD88, TRIF and MAVS, the cellular adaptor molecules involved in the primary TLR-RLR response to RNA virus infections, still control NiV infection, suggesting the engagement of cGAS/STING as an additional signalling pathway limiting paramyxovirus replication (Iampietro et al., 2020). Indeed, the ability to survive to NiV challenge is completely abolished in quadruple MyD88/TRIF/MAVS/STING KO mice, indicating a crucial and non-redundant role of STING axis in the response to NiV infection (Iampietro et al., 2021). Furthermore, the molecular evidence of STING activation was provided with the characterization of STING phosphorylation and K63-linked ubiquitination in both murine and human cellular models (Iampietro et al., 2021).

A recent study has demonstrated that MeV indirectly activates cGAS by inducing mitochondrial stress, with downregulation of mitochondrial biogenesis, hyperfusion of mitochondria and release of mtDNA in cytosol through a mitofusin 1 (Mfn1)-dependent mechanism (Sato et al., 2021) (**Figure 3**). Cytoplasmic mtDNA is then recognized by cGAS, triggering the activation of cGAS/STING signalling and IFNβ expression (Sato et al., 2021).

Contrasting results have been found regarding the role of STING during Sendai virus (SeV) infection, another paramyxovirus of *Respirovirus* genus. Whereas STING contributes to SeV restriction *in vitro*, STING phosphorylation and relocalization to Golgi do not occur during SeV infection (Franz et al., 2018). Moreover, a slight increase in IFNβ induction is detected in absence of STING, suggesting a possible proviral effect towards SeV (Franz et al., 2018). Adversely, studies performed on chicken cGAS (chcGAS) and STING (chSTING) report STING-dependent IFN-I production after SeV and VSV infection, since cells KO for chcGAS and chSTING express significantly lower amounts of IFNβ compared to WT cells (Cui et al., 2020; Li et al., 2020). Finally, a partially different result was obtained in human cells, as STING KO but not cGAS KO THP-1 cells elicit a significantly lower IFNβ expression compared to WT cells following SeV infection, suggesting a cGAS-independent role of hSTING on SeV restriction (Holm et al., 2016).

### 2.5 Other viral families

STING activation through diverse mechanisms has been reported following infections by additional viral families. The importance of STING in the response to *Togaviridae* infection is characterized by the fact that STING restricts Chikungunya virus (CHIKV) replication *in vivo* and SINV is inhibited by STING-dependent translation inhibition as described above (Franz et al., 2018; Geng et al., 2021). Moreover, IFI16 exerts an antiviral effect against CHIKV, as its deletion increases viral replication *in vitro* (Wichit et al., 2019). The ability of STING to suppress viral replication *in vitro* has also been demonstrated during *Arteriviridae* infection, particularly for porcine reproductive and respiratory syndrome virus (PRRSV) (Xu et al., 2021b). However, the mechanism mediating the activation of STING signalling during these viral infections remains unknown.

Vesicular stomatitis virus (VSV) replication is increased in absence of cGAS *in vivo*; indeed, cGAS/STING signalling is activated following *Rhabdoviridae* infection through two different non-canonical mechanisms (**Figure 3**). First, during VSV infection, the nuclear pool of cGAS interacts with protein arginine methyltransferase 5 (Prmt5), that demethylates histone H3 arginine 2 in correspondence of IFNβ and IFNα4 promoters (Cui et al., 2020). This modification facilitates the access of IRF3 in the nucleus and consequently the expression of IFN-I. Indeed, Prmt5 deficiency leads to the suppression of IFN-I response and increased susceptibility to viral infections (Cui et al., 2020). This discovery particularly highlights the importance of cGAS as nuclear protein, whose role goes beyond STING agonism as it actively modulates innate immunity through the regulation of chromatin structure. Also, STING-dependent translation inhibition acts on both viral and host proteins to restrict viral replication (Franz et al., 2018). In this context, no translocation of STING to Golgi is observed, suggesting that STING may perform translation inhibition by acting directly from the ER through an unknown strategy. Finally, STING inhibits translation through a RIG-I dependent mechanism, displaying a non-canonical mechanism of STING activation and an example of cross-talk between RNA- and DNA-dependent immune signalling pathways (Franz et al., 2018).

While STING, cGAS and IFI16 inhibit murine norovirus (MNV) replication in murine macrophages, STING also interacts with mouse RIG-I and inhibit its downstream signalling, suggesting that STING may exhibit both antiviral and proviral functions during *Caliciviridae* infections in mice (Yu et al., 2021). The activation of STING during MNV infections is dependent on leaked genomic and mitochondrial DNA, whose release in the cytoplasm appears to be associated to NS4 viral protein expression (Jahun et al., 2021) (**Figure 3**).

Finally, a particular mechanism for STING activation during *Bunyaviridae* infection has been recently highlighted. Following severe fever with thrombocytopenia syndrome virus (SFTSV) infection, viral RNA can be detected in the cytoplasm by nuclear matrix protein scaffold attachment factor A (SAFA) (Romig et al., 1992; Cao et al., 2019) (**Figure 3**). This sensor is responsible for viral RNA recognition in the nucleus; however, SFTSV nucleocapsid protein (NP) sequesters SAFA in the cytoplasm by interacting with and blocking the access to its nuclear localization signal (NLS) (Liu et al., 2021a). After the recognition of the viral genome in the cytosol, SAFA interacts with STING and stimulates its downstream signalling through TBK1 and IRF3 (Liu et al., 2021a). Interestingly, this study shows non-canonical mechanisms of action for both SAFA and STING. On one hand, SAFA was previously known to act in the nucleus by enhancing the antiviral response through chromatin remodeling, while during SFTSV infection it exerts its function in cytoplasm as STING agonist. On the other hand, this work represents a novel evidence of STING activation by RNA sensors, thus expanding the range of action of STING signaling against both DNA and RNA viruses. However, the reason why SFTSV developed such mechanism of SAFA antagonism leading to the non-canonical activation of STING remains unclear.

## 3 Immune evasion of STING signalling by RNA viruses

**Figure 4:**
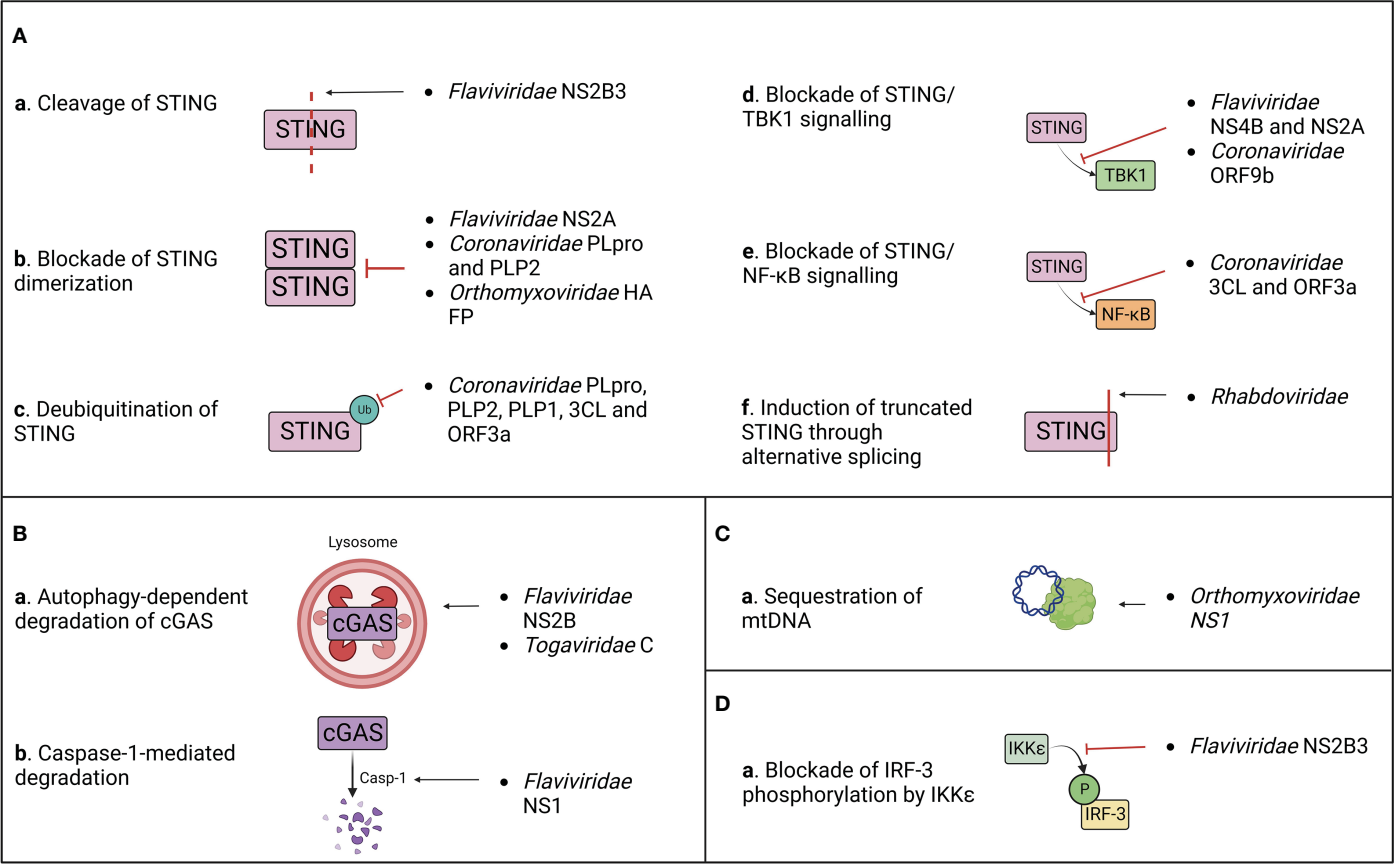
Summary of the known mechanisms of cGAS/STING antagonism by RNA viruses as described in (**Table 1**). Viral evasion strategies targeting STING **(A)**, cGAS **(B)**, mitochondrial DNA **(C)** or STING-dependent downstream signaling **(D)**.

Despite the well-known role of cGAS/STING axis in the immune response to DNA viruses, the first evidence of STING antagonism was observed in the context of a RNA virus infection by the flavivirus Dengue virus (DENV) (Maringer and Fernandez-Sesma, 2014). This discovery paved the way for the identification of STING evasion mechanism by several other RNA viruses belonging to different families (summarized in **Table 1**; **Figure 4**), underlying the importance of STING axis in the protection against RNA virus infections.

**Table 1:** RNA virus families exerting evasion strategies towards the cGAS/STING axis.

Viral Family	Viral species	Viral protein	Target	Mechanism
***Flaviviridae***	DENV, ZIKV, WNV, JEV	NS2B3 (Angleró-Rodríguez et al., 2014, 3)	hSTING	Direct cleavage of hSTING
			IKKϵ	Block of IKKϵ kinase activity and IRF-3 downstream signalling
	HCV, DENV, YFV	NS4B (Ding et al., 2013; Nitta et al., 2013)	STING	Disruption of STING-TBK1 or STING-MAVS interaction
	DENV	NS2B (Aguirre et al., 2017)	cGAS	Autophagy-lysosomal pathway-dependent degradation
	ZIKV	NS1 (Zheng et al., 2018, 1)	cGAS	Caspase-1 stabilization by K11-linked Ub removal
	DTMUV	NS2B3 (Wu et al., 2019)	duSTING	Direct cleavage of duSTING
		NS2A (Zhang et al., 2020)	duSTING	Binding of STING, block of TBK1 recruitment and activation, impairment of STING dimerization and signalling
***Coronaviridae***	SARS-CoV	PLpro (Sun et al., 2012)	STING	Block of STING dimerization through both direct mechanical interaction and viral DUB activity
	HCoV-NL63	PLP2 (Sun et al., 2012)	STING	Block of STING dimerization through both direct mechanical interaction and viral DUB activity
	SARS-CoV-2	3CL (Rui et al., 2021)	STING	Suppression of K63-linked STING ubiquitination and NF-κB signalling
		ORF3a (Rui et al., 2021)	STING	Suppression of K63-linked STING ubiquitination and NF-κB signalling
		ORF9b (Han et al., 2021)	STING	Direct binding of STING and suppression of TBK1/IRF3 signalling
	PEDV	PLP2 (Xing et al., 2013)	STING	Binding and suppression of K63-linked ubiquitination of STING and RIG-I; suppression of STING-dependent IFNβ expression
	TGEV	PLP1 (Hu et al., 2017)	STING	Suppression of STING and RIG-I ubiquitination
***Orthomyxoviridae***	IAV	HA FP (Holm et al., 2016)	STING	Direct interaction and block of STING dimerization
		NS1 (Moriyama et al., 2019)	mtDNA	Sequestration of cGAS/STING-activating mtDNA through IAV RNA binding domain
***Togaviridae***	CHIKV	C (Webb et al., 2020)	cGAS	IFNβ inhibition through early autophagy-dependent degradation of cGAS
		nsP1 (Webb et al., 2020)	STING	Binding of STING and stabilization of viral nsP1 protein
***Rhabdoviridae***	VSV	unknown factor (Rodríguez-García et al., 2018)	STING	Alternative splicing skewing towards truncated hSTING isoform over the WT isoform, causing the lack of IFNβ induction

### 3.1 Flaviviridae

After target cell penetration, viruses belonging to the *Flaviviridae* family adopt both passive and active strategies to evade the host immune response (Aguirre et al., 2012). On one hand, early viral proteins expression induces the rearrangement of ER membrane, generating vesicles that serve both as a micro-environment for viral replication and as a physical barrier to hide viral PAMPs from cellular PRRs (Peña and Harris, 2012; Rajah et al., 2020). On the other hand, flaviviruses actively target host immune effectors through their non-structural proteins to block antiviral signalling (Kao et al., 2018). This results in the inhibition of IFN-I production and in a defective activation of the adaptive immune response, which are both associated to the symptomatology observed in DENV, West Nile virus (WNV), Zika virus (ZIKV) and other flavivirus infections (Guo et al., 2005; Rodriguez-Madoz et al., 2010; Kumar et al., 2016). However, these viruses adopt different approaches to counteract the host immune surveillance (**Table 1**; **Figure 4**).

During DENV, ZIKV, WNV and Japanese encephalitis (JEV) infection, the inhibition of IFN-I production is partially attributed to the activity of the proteolytic core of NS2B3 viral protease complex, constituted by the last 40 aa of NS2B and the first 180 aa of NS3 non-structural proteins (Aguirre et al., 2012; Zhu and Fernandez-Sesma, 2020). Moreover, it has been characterized that DENV and ZIKV NS2B3 cleaves human STING (hSTING) but not its murine ortholog (mSTING or MPYS), which represents a species-specific restriction factor (Aguirre et al., 2012; Yu et al., 2012; Ding et al., 2018; Stabell et al., 2018). Interestingly, NS2B3 is also unable to cleave STING of non-human primates (NHP), and while clinical signs are observed following ZIKV infection in NHP, none appear during DENV infection (Ding et al., 2018; Stabell et al., 2018). Furthermore, conflicting results about the putative cleavage site of hSTING have been reported. Indeed, while it was previously shown that DENV NS2B3 cleaves human STING at arginine/glycine (RG) 95/96 in the transmembrane domain, later studies demonstrated that STING remains sensitive to cleavage after the mutation of RG 95/96 (Aguirre et al., 2012; Yu et al., 2012; Ding et al., 2018). In contrast, RG 78/79 residues located in hSTING cytoplasmic loop were identified as a novel cleavage site present only in hSTING, but not in mouse and NHP STING (Ding et al., 2018; Stabell et al., 2018). Nonetheless, the insertion of RG 78/79 cleavage site alone in mSTING does not rescue the STING cleavage pattern observed in humans, suggesting either the need of a full-length hSTING conformation for NS2B3 cleavage or the presence of additional murine restriction factors participating to the protection of STING (Ding et al., 2018; Zhu and Fernandez-Sesma, 2020). For this reason, the development of a transgenic mouse model where mSTING is replaced by hSTING could provide a useful tool to study *Flaviviridae* pathogenesis *in vivo* (Aguirre et al., 2012; Maringer and Fernandez-Sesma, 2014). Interestingly, the three main human STING haplotypes (RGRR, HARQ, and RGHR), based on four missense variations at residues 71, 230, 232 and 293 of hSTING and their combinations, display a different susceptibility to cleavage by NS2B3, HARQ haplotype being more prone to cleavage compared to the others, with possible consequences on individual predisposition to flavivirus-induced diseases (Zhu and Fernandez-Sesma, 2020). In addition to direct cleavage of STING, NS2B3 also dampens IFN-I expression downstream cGAS/STING pathway through direct interaction with the NTD of IKKϵ to block its kinase activity, thus preventing the phosphorylation of IRF-3 at its Ser386 and its further nuclear translocation (Angleró-Rodríguez et al., 2014). This antagonist activity is independent of the catalytic domain of the viral protease, suggesting that NS2B3 counteracts IFN-I induction through both catalysis dependent and independent mechanisms (Angleró-Rodríguez et al., 2014). A similar activity has been observed during duck Tembusu virus (DTMUV) infection, whose NS2B3 protein binds and cleaves duck STING (duSTING) (Wu et al., 2019). In parallel, DTMUV NS2A protein binds directly to STING thus competing with its interaction with TBK1, furthermore blocking TBK1 phosphorylation and subsequent STING activation (Zhang et al., 2020).

NS4B non-structural protein of Hepatitis C virus (HCV), DENV and Yellow Fever virus (YFV) targets STING, contributing to the inhibition of the host IFN-I response (Ding et al., 2013). However, even if NS4B interacts directly with STING, it doesn’t affect STING protein levels or oligomerization state. In contrast, NS4B has been demonstrated to counteract STING-related signalling by preventing the contact between STING and TBK1 (Ding et al., 2013) and/or by disrupting STING/MAVS interaction (Nitta et al., 2013), even though its mechanism of action is not clarified yet.

Besides STING, cGAS is also counted among the targets of flavivirus antagonism. DENV NS2B protease cofactor targets both human and mouse cGAS for degradation through an autophagy-lysosomal dependent pathway, preventing cGAS/STING signalling activation and downstream IFN-I production (Kao et al., 2018). This is corroborated by the fact that cGAS degradation is rescued by the administration of autophagy inhibitors (DBeQ, NH4Cl and 3-MA) and that cGAS colocalizes with autophagosomes and viral proteins during DENV infection (Aguirre et al., 2017). On the other hand, ZIKV adopts an indirect mechanism for cGAS degradation. ZIKV non-structural protein NS1 stabilizes caspase-1 by preventing its K11-linked ubiquitination and proteasomal degradation through the recruitment of deubiquitinase (DUB) USP8 (Wang et al., 2017) thus increasing its subsequent ability to cleave cGAS (Zheng et al., 2018).

### 3.2 Coronaviridae

After the discovery of the ability of flaviviruses to antagonize cGAS/STING axis, a novel strategy of immune evasion was attributed to another family of positive-stranded RNA viruses, the *Coronaviridae* (Sun et al., 2012) (**Table 1**; **Figure 4**). Indeed, a STING antagonizing activity was ascribed to SARS-CoV and HCoV-NL63 papain-like proteases (PLPs), contained within non-structural protein 3 (nsp3) of the coronavirus replicase complex (Sun et al., 2012). PLPs cleave the replicase polyprotein together with 3C-like protease (3CLpro), generating a variety of non-structural proteins (V’kovski et al., 2021). Non-structural proteins then interact with ER membrane, leading to the formation of double membrane vesicles (DMV) harbouring viral replication, while PLPs remain tethered to DMVs through their transmembrane domain (TM) (Mohan and Wollert, 2021). SARS-CoV and HCoV-NL63 PLPs (PLpro and PLP2, respectively) have developed the ability to antagonize STING signalling at different levels through both catalytic dependent and independent mechanisms (Sun et al., 2012). PLpro and PLP2 can indeed obstacle STING dimerization, either preventing its dimerization or by inducing the dissociation of STING dimers (Sun et al., 2012). This capacity could be partially due to the deubiquitinase (DUB) activity of PLPs, that inhibits K63-linked STING ubiquitination, and partially to a physical interaction between PLP-TM and STING, characterized by their colocalization in perinuclear puncta in virus-infected cells (Sun et al., 2012; Ma and Damania, 2016). This is further supported by the fact that catalytically inactive PLP mutants maintain their ability to inhibit STING ubiquitination, suggesting a steric block of STING access to cellular ubiquitinases (Sun et al., 2012). Consequently, the defective dimerization of STING leads to the lack of IRF3 nuclear translocation and subsequent IFN-I expression (Maringer and Fernandez-Sesma, 2014). A similar effect was observed for both PLP2 and PLP1 proteins of porcine epidemic diarrhea virus (PEDV) and transmissible gastroenteritis virus (TGEV), respectively, that exert DUB activity on both RIG-I and STING, thus inhibiting STING-dependent IFNβ expression (Xing et al., 2013; Hu et al., 2017).

Interestingly, a different mechanism of STING antagonism was observed during SARS-CoV-2 infection. First, the viral protease 3CL hampers cGAS/STING-mediated immune response by suppressing K63-linked ubiquitination of STING and subsequent NF-κB p65 nuclear translocation, but not IRF3 activation (Rui et al., 2021). This process could involve the enzymatic core of 3CL, since 3CL catalytic site mutants display a reduced ability to inhibit K63-linked STING ubiquitination (Rui et al., 2021). Moreover, the SARS-CoV-2 accessory protein ORF3a specifically prevents STING activation through direct interaction with both its N and C terminal domains (Rui et al., 2021). Similarly to 3CL, ORF3a has been shown to dampen the nuclear accumulation of p65 and its downstream gene expression, without affecting IRF3 translocation (Rui et al., 2021). A complementary activity was observed for the viral ORF9b, that binds STING directly, preventing TBK1-induced IRF3 activation and suppressing IFN-I expression through an uncharacterized mechanism (Han et al., 2021).

### 3.3 Influenza A virus (*Orthomyxoviridae*)

Even though the ability of evading the cGAS/STING axis was initially observed with ssRNA(+) viruses, the negative-sense ssRNA Influenza A virus (IAV) was discovered to antagonize STING signalling through both direct and indirect mechanisms (Holm et al., 2016; Moriyama et al., 2019) (**Table 1**; **Figure 4**). First, IAV targets STING directly through its hemagglutinin (HA) fusion peptide (FP), situated at the NTD of the HA2 subunit (Chen et al., 2018). Indeed, FP colocalizes with a small pool of STING in early endosomal compartments, leading to the blockade of STING dimerization and the inhibition of its downstream signalling (Holm et al., 2016). FP binds STING to its highly conserved residues 162-172, that overlap with both the STING dimerization region and the cGAMP binding pocket, thus being fundamental for STING activation and signal transduction (Holm et al., 2016). Interestingly, FP has been found to obstruct only fusion-induced STING activation and IFN-I expression, but not DNA/cGAS/cGAMP-induced STING activation, suggesting the presence of two alternative pathways of STING activation through DNA or through cell fusion (Holm et al., 2016; Abe et al., 2019). Moreover, the non-structural protein NS1 of influenza virus associates with mtDNA through its RNA binding domain, contributing to the evasion from STING-dependent antiviral immunity, triggered by mtDNA leakage in the cytoplasm following IAV infection (Moriyama et al., 2019).

### 3.4 Other viral families

In addition to the mechanisms of escape described above, other viral families developed alternative strategies to antagonize STING pathway, suggesting a wider involvement of STING in the response to RNA virus infections (**Table 1**; **Figure 4**). CHIKV suppresses IFNβ expression by inducing the autophagy-dependent degradation of cGAS as early as 4 h post infection (Webb et al., 2020). Interestingly, this process occurs in the presence of the viral capsid protein (C) alone, suggesting that C protein itself is further responsible for the degradation of cGAS. Moreover, nsP1 of CHIKV binds STING and is stabilized by this interaction (Webb et al., 2020). This suggests a dual proviral mechanism: on one hand, nsP1 could block STING function and signaling through its physical interaction and, at the same time, the virus would benefit from the stabilization of nsP1 protein, contributing to viral replication, transcription and particle assembly (Zhang et al., 2021).

Finally, *Rhabdoviridae* also evade STING response. VSV skews the alternative splicing of STING transcripts, resulting in the preferential translation of a truncated STING isoform that fails to induce IFNβ (Rodríguez-García et al., 2018). However, the mechanism and effectors of splicing dysregulation by VSV remain unknown and will require further investigations.

## 4 Antiviral approaches based on the modulation of STING/cGAS axis

The modulation of STING axis has emerged in recent years as a promising therapeutic strategy against cancers and autoimmune diseases. Several STING agonists have been tested or are currently under clinical trial due to their ability to boost immune response against tumor cells, while STING inhibitors have been proposed as a potential treatment against autoimmune disorders (Zhang et al., 2022b; Zhao et al., 2022). Due to its important role in the response to viral infections, STING represents an interesting target for antiviral therapies. This is sustained by the observation that the STING agonist DMXAA protects mice from mouse-adapted influenza viral strains (Shirey et al., 2011).

In the treatment of RNA virus infections direct or indirect STING agonists have been mainly tested as vaccine adjuvants. The polysaccharidic adjuvant chitosan potently enhances antigen-specific antibody production and T cell responses by inducing STING-dependent IFN I expression in dendritic cells (DC) *in vivo*, through the induction of mitochondrial and nuclear DNA release in cytoplasm (Carroll et al., 2016). Moreover, the intranasal delivery of cGAMP enclosed in biomimetic liposomes which mimic pulmonary surfactant enhances humoral and CD8+ T cell responses to influenza vaccine in mice and ferrets (Wang et al., 2020). Finally, the small molecule STING agonist CF501 has been proven to strongly enhance neutralizing antibodies production and T cell activation in NHP as adjuvant of a novel vaccine against sarbecoviruses, including the Delta variant of SARS-CoV-2 (Liu et al., 2022b).

As far as SARS-CoV-2 is concerned, conflicting results about the modulation of STING as COVID19 therapy have been found. The specific inhibitor of STING H151 reduces TNFα expression in infected cells, suggesting a possible involvement of STING in the aberrant activation of NF-κB and cytokine storm during SARS-CoV-2 infection (Neufeldt et al., 2020). In parallel, the diamidobenzimidazole-based STING agonist diABZI has been proven to be protective against SARS-CoV-2 infection *in vitro* and *in vivo* (Humphries et al., 2021; Li et al., 2021; Liu et al., 2021b; Zhu et al., 2021). This suggests that, during Sars-CoV-2 infection, STING may have a bivalent function: on one hand, it could lead to the worsening of COVID-19 condition in the case of its prolonged over-activation and subsequent excessive cytokines expression; on the other hand, it contributes to block viral replication and spreading through the induction of a robust IFN response.

Nonetheless, despite the large number of available STING agonists which have been already tested in type I, II and III clinical trials in the treatment of different type of tumours (Zhang et al., 2022b), no antiviral treatment targeting STING has reached clinical trials up to now. The main reason for this gap could be that, due to its ubiquitous expression and its strong effect on IFN activation, STING must be finely tuned to prevent unwanted detrimental effects, such as interferonopathies. In the development of STING-targeted antiviral therapeutics, three main issues must be considered: (a) The delivery should be possibly targeted to early infected cells to prevent systemic inflammation. Targeted delivery could be achieved through nanoparticle-based systems, as previously suggested in the context of cancer immunotherapy (Shae et al., 2019). (b) Timing: STING agonists could transiently boost IFN production and block viral spreading early after infections, but they could induce excessive IFN expression if administered too late and in a prolonged manner. In contrast, it could be speculated that it may be useful to deliver STING antagonists in those infections which are characterized by uncontrolled and/or chronic inflammation. (c) The infectious context: STING axis can respond in different manners to different pathogens, especially in the case of RNA viruses, which induce its activation through non-canonical mechanisms and simultaneously activate RLRs and TLRs which can synergize with cGAS/STING activation. Moreover, due to viral antagonism and cross-talk between immune signaling axes, the modulation of STING could produce unexpected effects leading to immunopathogenesis. Since the interaction between diverse signaling pathways in response to pathogens is finely tuned, particular care should be taken when targeting immune axes to avoid subverting this delicate balance. Finally, it must be taken into account that STING axis is involved in numerous physiological cellular processes, such as senescence and cell death, autophagy and translation regulation (Chen and Xu, 2022). For example, IRF3 activation by STING can result in intrinsic apoptosis activation, with opening of BCL-2 associated X protein/BCL-2 homologous antagonist/killer (Bax/Bak) pore and release of mtDNA in the cytoplasm, potentially leading to an amplification loop of STING activation (Liu and Guan, 2018). As a consequence, the inhibition or over-activation of STING even in an infectious setting could lead to the disruption of cell homeostasis with possible worsening of the pathology and risk of autoimmune or neurodegenerative diseases. Thus, it is fundamental to both clarify the molecular details about STING function in response to RNA virus infections and to develop novel therapeutic technologies in order to allow a controlled modulation of this potent IFN-inducing pathway.

## 5 Conclusion

cGAS/STING axis is known as the main innate immune pathway responsible for the recognition of cytosolic dsDNA of exogenous and/or endogenous origin (Ishikawa and Barber, 2008; Civril et al., 2013; Hopfner and Hornung, 2020). While its mechanism of action in response to bacteria, protozoa and DNA viruses is well deciphered, a contribution of this axis in the control of RNA virus infections has emerged in recent years. It was discovered that diverse families of enveloped RNA viruses antagonize STING through both direct and indirect strategies displayed over diverse structural and non-structural proteins (Aguirre et al., 2012; Sun et al., 2012; Holm et al., 2016). This ability revealed a necessity of RNA viruses to develop mechanisms to evade this immune signalling pathway, pointing out the importance of cGAS/STING against RNA virus infections (Aguirre et al., 2012; Sun et al., 2012; Holm et al., 2016). In addition, it was observed that cGAS/STING pathway is activated through indirect mechanisms, such as the induction of mitochondrial stress and chromatin/nuclear membrane damage, eventually resulting in the release of intracellular dsDNA in the cytoplasm and its subsequent mediation by cGAS or alternative DNA sensors (Chen et al., 2018; McGuckin Wuertz et al., 2019; Ren et al., 2021). Virus-induced cell membrane fusion, that is shared among numerous viral families, has emerged as an important cellular process bridging viral entry and the activation of STING through the damage and release of endogenous dsDNA induced by the disturbance of intracellular ion balance, oxidative stress and/or inflammation (Holm et al., 2012; Ku et al., 2020). Nevertheless, while the knowledge of RNA viruses-cGAS/STING signalling interplays has significantly improved in recent years, several issues regarding specific mechanisms of activation of this pathway following RNA virus infections remain obscure. Further insights will bring a more detailed understanding of RNA viruses’ immunopathogenesis and DNA-dependent response during RNA virus infections and help in the development of novel STING-based therapeutic approaches.

## Author contributions

LA wrote the manuscript, edited the manuscript and prepared the figures. BH and MI edited the manuscript and edited the figures. All authors contributed to the article and approved the submitted version.
